# Does time taken by paediatric critical care transport teams to reach the bedside of critically ill children affect survival? A retrospective cohort study from England and Wales

**DOI:** 10.1186/s12887-020-02195-6

**Published:** 2020-06-19

**Authors:** Sarah E. Seaton, Padmanabhan Ramnarayan, Patrick Davies, Emma Hudson, Stephen Morris, Christina Pagel, Fatemah Rajah, Jo Wray, Elizabeth S. Draper, on behalf of the DEPICT Study Team

**Affiliations:** 1 Department of Health Sciences, University of Leicester, University Road, Leicester, UK; 2 Children’s Acute Transport Service (CATS), Great Ormond Street Hospital NHS Foundation Trust, London, UK; 3 Respiratory, Critical Care and Anaesthesia Section, Infection, Immunity and Inflammation Research & Teaching Department, UCL GOS Institute of Child Health, London, UK; 4 Nottingham University Hospital NHS Trust, Nottingham, UK; 5 Department of Public Health and Primary Care, University of Cambridge, Cambridge, UK; 6 Clinical Operational Research Unit, University College London, London, UK; 7 Yorkshire and Humber Infant and Children’s Transport Service (Embrace), Barnsley, UK; 8 Heart and Lung Directorate, Great Ormond Street Hospital for Children NHS Foundation Trust, London, UK

**Keywords:** Paediatric intensive care, Paediatric transport, Critical care transport

## Abstract

**Background:**

Reaching the bedside of a critically ill child within three hours of agreeing the child requires intensive care is a key target for Paediatric Critical Care Transport teams (PCCTs) to achieve in the United Kingdom. Whilst timely access to specialist care is necessary for these children, it is unknown to what extent time taken for the PCCT to arrive at the bedside affects clinical outcome.

**Methods:**

Data from transports of critically ill children who were admitted to Paediatric Intensive Care Units (PICUs) in England and Wales from 1 January 2014 to 31 December 2016 were extracted from the Paediatric Intensive Care Audit Network (PICANet) and linked with adult critical care data and Office for National Statistics mortality data. Logistic regression models, adjusted for pre-specified confounders, were fitted to investigate the impact of time-to-bedside on mortality within 30 days of admission and other key time points. Negative binomial models were used to investigate the impact of time-to-bedside on PICU length of stay and duration of invasive ventilation.

**Results:**

There were 9116 children transported during the study period, and 645 (7.1%) died within 30 days of PICU admission. There was no evidence that 30-day mortality changed as time-to-bedside increased. A similar relationship was seen for mortality at other pre-selected time points. In children who waited longer for a team to arrive, there was limited evidence of a small increase in PICU length of stay (expected number of days increased from: 7.17 to 7.58).

**Conclusion:**

There is no evidence that reducing the time-to-bedside target for PCCTs will improve the survival of critically ill children. A shorter time to bedside may be associated with a small reduction in PICU length of stay.

## Background

Paediatric intensive care units (PICUs) were centralised in the United Kingdom (UK) in the 1990s, following international evidence and expert opinion that suggested improved risk-adjusted outcomes for critically ill children associated with centralisation of intensive care services [1–4]. As sick children may initially present with acute illness or injury to hospitals without a PICU, specialist Paediatric Critical Care Transport teams (PCCTs) were established as ‘mobile’ intensive care units to stabilise and safely transport them [5]. Currently, 25 PICUs and 10 PCCTs exist in England and Wales, covering a child population of 12 million, with approximately 16,000 PICU annual admissions [6], of which one-third to one-half are transported [6, 7]. Use of PCCTs for inter-hospital transport of critically ill children, rather than non-specialist teams, is associated with improved survival and there is no suggestion that travelling further is associated with poorer outcomes [7–9].

The population and geography served by each PCCT, as well as the varied workload of the team, means that median time to reach a sick child’s bedside (time-to-bedside) from acceptance of a referral in England and Wales varies between 50 and 140 min [6]. These teams initiate any critical care interventions such as invasive ventilation, insertion of chest drains and vasoactive drug infusions not already provided.

National quality standards from the UK Paediatric Intensive Care Society (PICS) state that PCCTs should reach the child’s bedside within three hours of agreeing that the child requires intensive care [10]. The National Health Service (NHS) England quality dashboard reports how frequently teams depart their base within 30 min of accepting a child for transport [11]. Similar targets have been adopted as quality metrics in international benchmarking initiatives [12, 13]. However, since acute hospitals may have initiated critical care interventions before the arrival of the PCCT, timely arrival of a PCCT may not be the main determinant of outcome. Centralisation of routine paediatric surgery and anaesthesia has led to increasing unfamiliarity of adult anaesthetic and intensive care teams with stabilisation of children [14], so more ambitious time standards for PCCTs may lead to further improvement in patient outcomes.

The DEPICT Study (Differences in access to Emergency Paediatric Intensive Care and care during Transport) is a national mixed-methods study investigating how differences in the timeliness of access to a PCCT and aspects of care provided by PCCTs during transport to PICU affect outcomes and experience for critically ill children and their families [15]. In this paper, we present the results of the primary statistical analysis of a large, high-quality linked dataset examining the impact of time-to-bedside on 30-day mortality (primary outcome) and other clinically relevant secondary outcomes.

## Methods

### Study population

The DEPICT cohort comprised all emergency (non-elective) transports of children under 16 years old undertaken by a PCCT and admitted to an NHS PICU in England and Wales between 1 January 2014 and 31 December 2016.

### Outcomes

The primary outcome was mortality within 30 days of PICU admission. Secondary outcomes related to mortality were: death on the PICU, death within two days of admission, 90 days and one year of admission to PICU. Secondary outcomes related to healthcare resource use were length of PICU stay (LOS) and length of invasive ventilation (LOV) on the PICU.

### Data sources

Information about children transported by a PCCT to PICU were extracted from the Paediatric Intensive Care Audit Network (PICANet, https://www.picanet.org.uk/) which collects data related to the referral, transport and admission of every child requiring admission to a PICU. Transports undertaken by neonatal transport teams were not considered. Data entry into PICANet within three months of the child’s discharge is recommended by PICS [10] and data completeness, including for NHS number which was used for the linkage, is around 99% [6]. PICANet uses a bespoke web-based data entry system supplemented by validation visits to ensure data are accurately entered from medical notes.

Information about admissions to a general (adult) intensive care unit (GICU) prior to transport to a PICU was provided by the Intensive Care National Audit and Research Centre (ICNARC) and linked to PICANet data using personally identifiable data by NHS Digital (https://digital.nhs.uk/). Mortality outcomes were provided from Office for National Statistics. Further details about the data flow and linkage can be found in the DEPICT Study protocol [15].

### Inclusion and exclusion criteria

Children were included if it was possible to link their PICANet transport record to the corresponding PICU admission. Children with missing referral data were excluded. If a child was transported multiple times during DEPICT we only included their final transport. Children were also excluded if there was missing information about ventilation status at referral, or if it was not possible to calculate time-to-bedside. Time-to-bedside is the difference between the time it was agreed that the child required transport to PICU and the time the team arrived at the child’s bedside. For the secondary outcomes of LOS and LOV exclusions were made if there was missing data.

### Statistical analysis

A statistical analysis plan was finalised prior to any analysis [15]. Summary statistics were reported as counts/percentages for categorical variables or median/range or mean/standard deviation for continuous variables. To investigate the impact of time-to-bedside on mortality, logistic regression models with clustered standard errors for the PCCT were fitted. Key confounders were selected a priori via discussion with the clinical members of the Study Management Group [15] and were: age of the child; Paediatric Index Mortality 2 score (PIM2) [16]; clinical diagnosis (based on PICANet diagnostic groups [6]); ventilation status at referral (yes/no not indicated/no advised to intubate); number of transport requests from the collection hospital during the study (categorised as < 50 requests;50 to < 100 requests;100+ requests) and whether the child was receiving critical care around the time of the transport request (collected from intensive care or receiving care in a GICU in the two days preceding transport, yes/no). Variables were included regardless of whether there was an association with mortality. Time-to-bedside was categorised as: ≤60 min; 61 to 90 min; 91 to 120 min; 121 to 180 min and 181+ minutes. Odds ratios and 95% confidence intervals were estimated alongside the (adjusted) probability of mortality by time-to-bedside. The Directed Acyclic Graph (DAG) is available via DAGgity (http://www.dagitty.net/) at: dagitty.net/mbDnLfo.

Clinical subgroups of children were selected a priori for investigation: cardiac/neurological conditions; low/high PIM2 score (low: PIM2 ≤ 0.10 and high: PIM2 > 0.10) and transport in summer/winter (summer: June/July/August, winter: December/January/February).

The performance of the model in the primary analysis was assessed using the Area Under the Curve (AUC) [17]; Hosmer Lemeshow test [18] and Briers Score [19]. Sensitivity analyses were performed to investigate the impact of using the final transport for children transported multiple times. The analysis was also repeated identifying those children who were transported multiple times in the study time window. Finally, the impact of missing data was investigated by re-fitting the model with different scenarios for the missing data.

Logistic models were also fitted with secondary mortality endpoints of: death on PICU; death within two days; death within 90 days and death within a year following PICU admission.

PICU LOS was calculated as the discharge date minus the admission date plus one. PICU LOV was the total days when a child received invasive ventilation for any part of the day. The outcomes of LOS and LOV were highly skewed (most children had a short LOS/LOV) and therefore negative binomial models were used with the same adjustments as the primary analysis. The expected (adjusted) LOS and LOV were estimated and presented graphically by time-to-bedside, alongside incidence rate ratios.

*P*-values are not reported in line with the DEPICT Study protocol, and emphasis is on trends and clinical significance [15].

### Ethical approval

DEPICT has ethical approval from the Health Research Authority, the National Research Ethics Service (London Riverside, reference: 17/LO/1267) and the Confidentiality Advisory Group (reference: [17] CAG0129).

## Results

### Study population

**Fig. 1:**
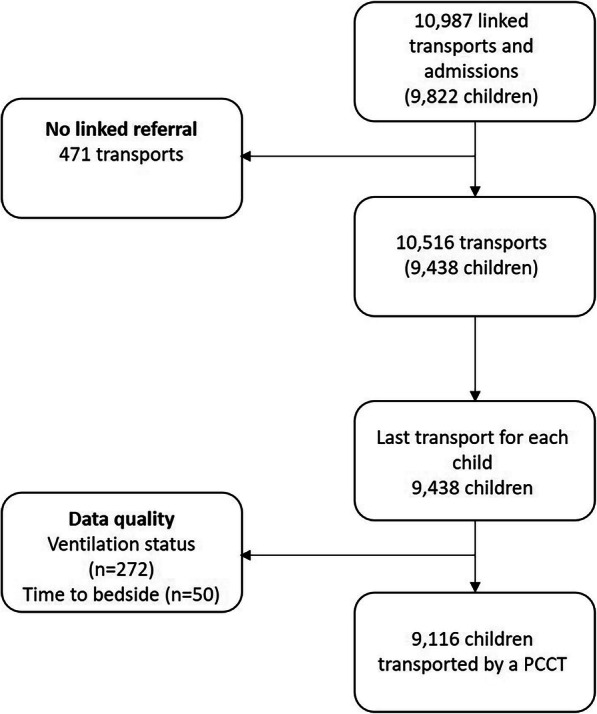
Flow chart for the primary analysis

There were 10,987 emergency transports by a PCCT of children aged under 16 years with a linked admission record to a PICU during the study (Fig. 1). Linkage between PICANet transport and admission records was very high (~ 97%). Transports not linked with a corresponding referral event were excluded (*n* = 471, 4.3%) leaving 10,516 transports. For children with multiple transports we used the latest transport, providing 9438 transported children. Children whose ventilation status at the time of referral was missing (*n* = 272) and those with missing or implausible data (defined as > 24 h) for the time-to-bedside (*n* = 50) were excluded, leaving 9116 children in the primary analysis.

Summary statistics are provided in Table 1 and separated into time-to-bedside: ≤60 min; 61 to 180 min or 181+ minutes. Just over half of children were under one year old and the most common diagnosis was respiratory problems (Table 1). At the time of referral, more than half of the children were already intubated or advice was given to intubate. In 393 of the 424 children (92.7%, Table 1) with the highest PIM2 score category, the PCCT arrived in less than three hours. Similarly, for the 338 children with a diagnosis of trauma, the transport team arrived in less than three hours 90% of the time (*n* = 305, Table 1).

**Table 1:** Characteristics of the children and transports included in the primary analysis (*n* = 9116)

Characteristic	Total	Arrived at the bedside in ≤ 60 min (***n*** = 2654)	Arrived at the bedside in 61 to 180 min (***n*** = 5271)	Arrived at the bedside in 181+ minutes (***n*** = 1191)
**Age, n (%)**				
< 1 year	4669 (51.2)	1371 (51.7)	2685 (50.9)	613 (51.5)
1 to < 5 years	2438 (26.7)	682 (25.7)	1437 (27.3)	319 (26.8)
5 to < 11 years	1174 (12.9)	344 (13.0)	679 (12.9)	151 (12.7)
11 to < 16 years	835 (9.2)	257 (9.7)	470 (8.9)	108 (9.1)
**Sex of child, n (%)**				
Male	5183 (56.9)	1552 (58.5)	2962 (56.2)	669 (56.2)
Female	3932 (43.1)	1102 (41.5)	2308 (43.8)	522 (43.8)
Unknown	1 (< 0.5)	0	1 (< 0.1)	0
**PIM2 score, n (%)**				
< 1%	1039 (11.4)	314 (11.8)	625 (11.9)	100 (8.4)
1 to < 5%	4089 (44.9)	1104 (41.6)	2409 (45.7)	576 (48.4)
5 to < 15%	2985 (32.7)	845 (31.8)	1710 (32.4)	430 (36.1)
15 to < 30%	579 (6.4)	220 (8.3)	305 (5.8)	54 (4.5)
30 + %	424 (4.7)	171 (6.4)	222 (4.2)	31 (2.6)
**Length of stay in PICU (days), median (10th, 90th)**	5 (2, 14)	5 (2, 15)	5 (2, 14)	5 (2, 15)
**Length of stay in PICU (days), mean (standard deviation)**	7.5 (13.2)	7.5 (15.1)	7.4 (11.6)	8.2 (15.2)
**Child received multiple transports during the time window of DEPICT, n (%)**	775 (8.5)	206 (7.8)	459 (8.7)	110 (9.2)
**Parent accompanied the child in the ambulance**				
Yes	6974 (76.5)	2188 (82.4)	3966 (75.2)	820 (68.9)
No, parent not present	432 (4.7)	135 (5.1)	237 (4.5)	60 (5.0)
No, parent declined to accompany	1150 (12.6)	233 (8.8)	713 (13.5)	204 (17.1)
No, parent not permitted to accompany	385 (4.2)	41 (1.5)	259 (4.9)	85 (7.1)
Unknown	175 (1.9)	57 (2.2)	96 (1.8)	22 (1.9)
**Collection area, n (%)**				
PICU	259 (2.8)	88 (3.3)	127 (2.4)	44 (3.7)
GICU	731 (8.0)	45 (1.7)	430 (8.2)	256 (21.5)
NICU	822 (9.0)	332 (12.5)	374 (7.1)	116 (9.7)
Theatre/Recovery and Theatre	1978 (21.7)	392 (14.8)	1328 (25.2)	258 (21.7)
X-ray/CT/Endoscopy/A&E	2773 (30.4)	1084 (40.8)	1464 (27.8)	225 (18.9)
Ward/HDU/Other intermediate area	2536 (27.8)	710 (26.8)	1539 (29.2)	287 (24.1)
Other/unknown	17 (0.2)	3 (0.1)	9 (0.2)	5 (0.4)
**Diagnostic group, n (%)**				
Respiratory	4355 (47.8)	1102 (41.5)	2591 (49.2)	662 (55.6)
Cardiovascular	1310 (14.4)	477 (18.0)	681 (12.9)	152 (12.8)
Endocrine	219 (2.4)	65 (2.5)	133 (2.5)	21 (1.8)
Haem/oncology	153 (1.7)	56 (2.1)	78 (1.5)	19 (1.6)
Infection	820 (9.0)	261 (9.8)	484 (9.2)	75 (6.3)
Neurological	1505 (16.5)	403 (15.2)	907 (17.2)	195 (16.4)
Trauma & accidents	338 (3.7)	121 (4.6)	184 (3.5)	33 (2.8)
Other	416 (4.6)	169 (6.4)	213 (4.0)	34 (2.9)
**Ventilated at time of referral call, n (%)**				
Yes	3814 (41.8)	1129 (42.5)	2109 (40.0)	576 (48.6)
No (not indicated)	2886 (31.7)	911 (34.3)	1652 (31.1)	323 (27.1)
No (advised to intubate)	2416 (26.5)	614 (23.1)	1510 (28.7)	292 (24.5)
**Size of acute hospital (based on transport requests in DEPICT time window), n (%)**				
Small (< 50 requests)	2274 (25.0)	635 (23.9)	1308 (24.8)	331 (27.8)
Medium (50- < 100 requests)	3802 (41.7)	1118 (42.1)	2129 (40.1)	555 (46.6)
Large (100+ requests)	3040 (33.4)	901 (34.0)	1834 (34.8)	305 (25.6)
**Receiving care in a critical care setting at collection organisation, n (%)**				
Yes	1951 (21.4)	479 (18.1)	1022 (19.4)	450 (37.8)
No	7165 (78.6)	2175 (82.0)	4249 (80.6)	741 (62.2)
**Mortality, n (%)**				
Died within two days of admission	278 (3.1)	105 (4.0)	153 (2.9)	20 (1.7)
Died in PICU	571 (6.3)	200 (7.5)	316 (6.0)	55 (4.6)
Died within 30 days of admission	645 (7.1)	226 (8.5)	357 (6.8)	62 (5.2)
Died within one year of admission	949 (10.4)	331 (12.5)	520 (9.9)	98 (8.2)

### Time-to-bedside and mortality

The majority of children were reached within three hours (~ 87%, Additional file 1: Figure 1) and few waited longer than six hours for a team to arrive (*n* = 216, 2.4%). Whilst we planned to exclude any time > 24 h, the maximum time-to-bedside was ~ 21 h.

**Fig. 2:**
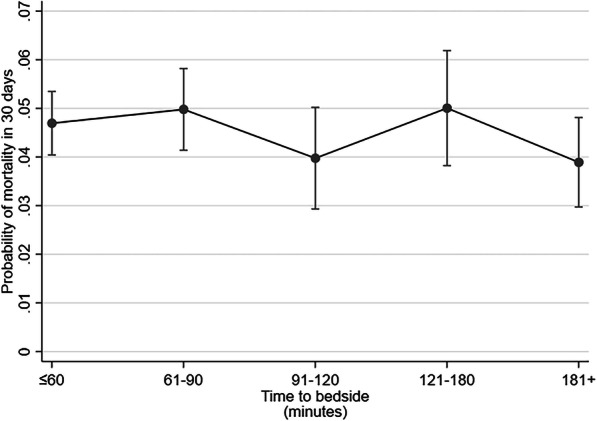
Probability of mortality within 30 days of PICU admission by time taken to reach the bedside whilst holding other variables in the model at the mean value

After adjustment, there was no evidence that time-to-bedside impacted on the odds of mortality 30 days after PICU admission (Table 2). All confidence intervals for time-to-bedside contained the point of no difference (odds ratio of one) and there was no suggestion of an increasing or decreasing trend of the probability of mortality against time-to-bedside (Fig. 2). Similar findings were seen for secondary mortality end-points of death in the PICU and at two days, 90 days and one year (Additional file 2: Figure 2).

**Table 2:** Multivariable analyses of the association between time taken to arrive at the bedside and mortality within 30 days admission in children transported by PCCT in England and Wales, accounting for characteristics of the child and their sickness (*n* = 9116)

Characteristic	Odds ratio (mortality in 30 days)	95% confidence interval (30 days)
**Time to arrive at bedside**		
≤ 60 min	Baseline	Baseline
61 to 90 min	1.06	0.87 to 1.31
91 to 120 min	0.84	0.66 to 1.08
121 to 180 min	1.07	0.91 to 1.26
181+ minutes	0.82	0.66 to 1.02
**Age**		
< 1 year	Baseline	Baseline
1 to < 5 years	0.96	0.79 to 1.16
5 to < 11 years	1.40	1.11 to 1.77
11 to < 16 years	1.24	0.94 to 1.64
**PIM 2**		
< 1%	Baseline	Baseline
1 to < 5%	2.22	1.17 to 4.23
5 to < 15%	3.61	1.98 to 6.60
15 to < 30%	11.31	5.77 to 22.19
30 + %	34.47	18.22 to 65.20
**Diagnosis**		
Respiratory	Baseline	Baseline
Cardiovascular	2.41	1.62 to 3.57
Endocrine	2.73	1.85 to 4.05
Haem/oncology	2.59	1.26 to 5.33
Infection	1.73	1.22 to 2.47
Neurological	1.28	0.76 to 2.16
Trauma and accidents	1.31	0.94 to 1.83
Other	1.81	0.96 to 3.44
**Ventilated at referral**		
No (not indicated)	Baseline	Baseline
Yes	1.37	1.19 to 1.57
No (advised to intubate)	0.94	0.79 to 1.12
**Collection unit size**		
Small	Baseline	Baseline
Medium	1.12	1.01 to 1.24
Large	1.04	0.88 to 1.23
**Receiving critical care**		
No	Baseline	Baseline
Yes	1.06	0.90 to 1.25

Cluster term is included in the model for the transport organisation, this adjusts the standard errors accordingly

The model performance for the primary analysis was good with AUC: 0.79, Hosmer-Lemeshow test *p* = 0.86 and Briers score of 0.056 indicating good model fit. The impact of missing data was investigated for the primary outcome by re-fitting the models including children excluded due to missing information about ventilation at the time of referral (*n* = 272, Fig. 1). The model was re-fitted to check the three scenarios where all missing data was related to children who were not intubated; to children who were intubated; and then children where there was advice given to intubate. The conclusions remained unchanged (not presented) for the impact of time-to-bedside on mortality. The model was also re-fitted using the first transport for children who were transported multiple times, and our conclusions remained unchanged (not presented). Similarly, when we included an indicator term for those children who had been transported multiple times and our conclusions around time-to-bedside remained unchanged (not presented).

The impact of time-to-bedside on mortality within 30 days of PICU admission was investigated for pre-selected subgroups of: cardiac/neurological conditions; low/high PIM2 score and transports in summer/winter. The sample size in each sub-analysis was reduced, and thus our power was reduced, but a lack of trend similar to those in the primary analysis was observed (Additional file 3: Figure 3).

### Time-to-bedside and length of stay (LOS)/length of ventilation (LOV)

**Fig. 3:**
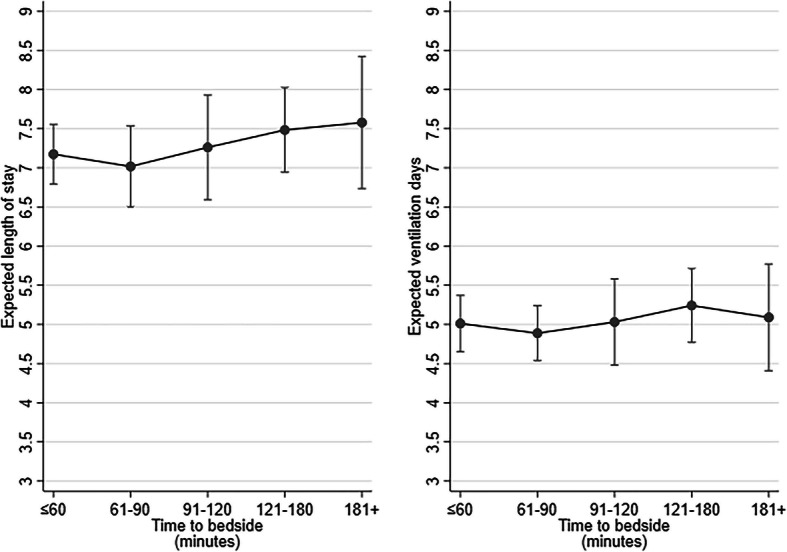
Expected PICU length of stay and length of ventilation in PICU (days) by time taken to arrive at the bedside whilst holding other variables in the model at the mean value

Children were excluded if data were missing related to LOS or LOV (LOS: *n* = 0, LOV: *n* = 1). The incidence rate ratios are provided in Table 3 and the expected LOS and LOV against time-to-bedside is provided (Fig. 3). For LOS there was slight evidence of an increasing trend as time-to-bedside increased with the expected LOS increasing from 7.17 to 7.58 days. There was no apparent trend for LOV with the expected number of days remaining around five days for all groups of time-to-bedside. Similar trends were observed when we conducted a sensitivity analysis by excluding children who died in PICU from the LOS/LOV analysis.

**Table 3:** Multivariable analyses of the association between time taken to arrive at the bedside and LOS in PICU (*n* = 9116) or LOV in PICU (*n* = 9115), given the child’s characteristics and sickness

Characteristic	Incidence rate ratio (LOS)	95% confidence interval (LOS)	Incidence rate ratio (LOV)	95% confidence interval (LOV)
**Time to arrive at bedside**				
≤ 60 min	Baseline	Baseline	Baseline	Baseline
61 to 90 min	0.98	0.90 to 1.06	0.98	0.90 to 1.06
91 to 120 min	1.01	0.92 to 1.12	1.00	0.91 to 1.11
121 to 180 min	1.04	0.96 to 1.13	1.05	0.97 to 1.13
181+ minutes	1.05	0.94 to 1.19	1.02	0.90 to 1.15
**Age**				
< 1 year	Baseline	Baseline	Baseline	Baseline
1 to < 5 years	0.93	0.84 to 1.02	0.87	0.78 to 0.98
5 to < 11 years	0.97	0.89 to 1.07	0.83	0.73 to 0.94
11 to < 16 years	0.96	0.83 to 1.10	0.79	0.64 to 0.99
**PIM 2**				
< 1%	Baseline	Baseline	Baseline	Baseline
1 to < 5%	1.24	1.14 to 1.36	1.32	1.18 to 1.48
5 to < 15%	1.62	1.52 to 1.72	1.82	1.68 to 1.97
15 to < 30%	2.08	1.80 to 2.39	2.40	2.03 to 2.84
30 + %	1.67	1.37 to 2.03	2.13	1.69 to 2.68
**Diagnosis**				
Respiratory	Baseline	Baseline	Baseline	Baseline
Cardiovascular	0.93	0.79 to 1.10	0.77	0.64 to 0.92
Endocrine	0.69	0.48 to 0.99	0.58	0.34 to 0.99
Haem/oncology	0.94	0.55 to 1.62	0.95	0.43 to 2.09
Infection	0.73	0.65 to 0.82	0.72	0.60 to 0.86
Neurological	0.56	0.47 to 0.67	0.54	0.44 to 0.66
Trauma and accidents	0.64	0.39 to 1.04	0.65	0.35 to 1.20
Other	0.83	0.67 to 1.04	0.83	0.60 to 1.14
**Ventilated at referral**				
No (not indicated)	Baseline	Baseline	Baseline	Baseline
Yes	0.98	0.89 to 1.08	1.19	1.05 to 1.35
No (advised to intubate)	0.94	0.85 to 1.05	1.11	1.02 to 1.21
**Collection unit size**				
Small	Baseline	Baseline	Baseline	Baseline
Medium	0.99	0.92 to 1.08	0.97	0.88 to 1.07
Large	0.99	0.92 to 1.07	1.00	0.92 to 1.08
**Receiving critical care**				
No	Baseline	Baseline	Baseline	Baseline
Yes	1.09	0.98 to 1.21	1.06	0.91 to 1.24

Cluster term is included in the model for the transport organisation, this adjusts the standard errors accordingly

## Discussion

The optimal trade-off between centralisation and rapid access to specialist care has been a topic of debate. Provision of early, high-quality care is known to improve outcomes in paediatric sepsis and head trauma [20, 21]. In a single-centre study from Canada, critically ill children transported, by non-specialist teams, from remote hospitals (> 350 km) had longer PICU and hospital LOS [22]. In contrast, distance from PICU did not affect risk-adjusted outcomes in studies where a specialist team was used for transport [7, 8], suggesting that initiation of critical care by PCCTs as soon as they reach the acute hospital may be beneficial. Therefore, it may appear reasonable that earlier arrival of a PCCT may be associated with improved survival.

Centralisation of inter-hospital transport allows for the concentration of expertise and specialist skills, but potentially makes reaching remote hospitals quickly more difficult. In the absence of robust evidence of the impact of centralisation on patient outcomes from either the UK or other international populations, current quality standards for paediatric critical care transport in the UK are based on expert opinion [10]. In this large retrospective cohort study our primary analysis demonstrated that there was no suggestion that 30-day mortality was associated with time-to-bedside, at least for times below six hours. This finding was replicated for mortality at other time points and in pre-defined subgroups. However, we also noted that there were few children with very high PIM scores who waited a long time for a team to arrive at their bedside. We observed limited evidence of a trend between increasing time-to-bedside and longer PICU LOS.

Our findings have implications for critical care services. First, time-to-bedside targets are met by PCCTs in the majority of cases, particularly for the sickest children [5, 6]. Second, although we are unable to definitely conclude that the current time-to-bedside target can be relaxed further due to the small numbers of children waiting longer than three hours for a PCCT, we can more confidently suggest that reducing the time target further (e.g. to 1 or 2 h) would not confer a survival benefit, and would involve significant service reconfiguration [23]. Previous studies have shown that UK hospitals perform a large proportion of critical care interventions before the arrival of the PCCT with the support of general (adult) anaesthetic and critical care teams [14] and this may be different in other countries. Children are also occasionally cared for in GICUs while awaiting the PCCT. In our cohort, 1951 children were being cared for in an intensive care setting prior to the arrival of the PCCT, and of these ~ 40% were admitted to GICUs prior to transport, although considerable regional variation exists in this practice [24]. Third, since PICU LOS increased with longer time-to-bedside, the main impact of early arrival of PCCTs, and associated greater physiological stabilisation prior to PICU admission, may be to shorten the time it takes for the child to recover from critical illness. The observed average reduction of half a day in PICU LOS should be considered alongside the fact that median PICU LOS was only five days (mean: 7.5 days).

### Strengths and limitations

The UK is unique internationally in terms of setting national standards for PCCTs and collecting robust transport data through national audit. Our study is therefore the first large scale nationwide study to investigate the impact of time-to-bedside on a critically ill child. The data for this study were from a linked dataset of routine data and whilst there are limitations of using routine data, notably quality and completeness, PICANet is a large national clinical audit collecting data from all UK PICUs with a published ascertainment rate > 99% [6]. The level of missing data in this study was very low and sensitivity analyses indicated the impact was minimal. It is likely that the quality of some variables, such as the time when it was agreed the child required paediatric intensive care, may have improved over time. Whilst we selected key confounders carefully, residual confounding may still exist. This may be particularly apparent in the use of the PIM2 score, which may not completely capture the severity of the child’s condition. In this work we only considered children who were transported to PICU and did not compare with a control group of children who did not require transport.

We selected 30-day mortality as the primary outcome as this captured deaths in PICU as well as those occurring in other settings including hospices. Whilst it is unlikely that deaths occurring several days after transport will be related to the time-to-bedside, we saw similar results with mortality in shorter timeframes. This work excluded deaths before admission to PICU following a request for transport, although the occurrence of this is low (< 1%) [6]. Occasionally children referred for transport by a PCCT are refused due to being time-critical and we were unable to investigate this group which may have poorer outcomes [6]. PICANet does not collect data on long-term outcomes such as functional status, so it was not possible to investigate this.

Our work has investigated transports in England and Wales, which may be different to other countries, particularly in terms of skills available at hospitals who provide the initial care. Availability of staff trained in advanced paediatric life support during stabilisation of sick children may be one factor that may vary across healthcare systems and potentially affect outcome. Therefore, in other settings, time-to-bedside may matter more, although currently there is no international dataset of paediatric transports of children who require intensive care. Similarly, distance travelled to PICU may be less in the UK than other countries, and the vast majority of transports in England and Wales (> 95%) are undertaken by road ambulance rather than fixed wing or helicopter [6].

Finally, the three-hour target may minimise the time local hospitals have to care for critically ill children, potentially impacting on care provision of other patients; the staff experience, as well as the experience of parents waiting for the PCCT to arrive, is being investigated in another DEPICT workstream.

### Future work

In this study we only considered children who were transported to PICU and in future work we plan to compare these children with those who were admitted directly to PICU. We also plan to investigate the impact of the time taken for a child to reach the PICU, and the critical care interventions they receive from the local hospital and the PCCT before and during transport. Previous research has suggested that stabilisation time does not impact on patient outcome and a larger definitive study needs to investigate this further [25]. Other workstreams from DEPICT will report on the experiences of children and their families, as well as staff members [15].

## Conclusion

Time-to-bedside has been a key target for PCCTs to achieve in recent years. This work has demonstrated that there was no association with increased or reduced mortality as time-to-bedside increased. Waiting longer than three hours was unusual and so it was not possible to assess if the target could be relaxed further. This suggests that maintaining the current target to reach the child’s bedside is appropriate at this time, although this should be considered in the future alongside parent experience and staff perspectives.

## Supplementary information

**Additional file 1: Figure 1:** Distribution of time taken to reach the bedside in the hospital requesting paediatric transport (*n* = 9116).

**Additional file 2: Figure 2:** Mortality in the PICU, in two days, 90 days and within one year of admission against time taken to reach the bedside whilst holding other variables in the model at the mean value.

**Additional file 3: Figure 3:** Subgroup analysis for time taken to reach the bedside on mortality within 30 days of admission to PICU. Adjustments are as in the primary analysis.

## Data Availability

Data used in this study can be requested directly from NHS Digital, PICANet and ICNARC.

## Abbreviations

PICU: Paediatric intensive care unit
PCCT: Paediatric Critical Care Transport team
PICS: Paediatric Intensive Care Society
NHS: National Health Service
LOS: Length of PICU stay
LOV: Length of invasive ventilation on the PICU
PICANet: Paediatric Intensive Care Audit Network
GICU: General Intensive Care Unit
ICNARC: Intensive Care National Audit and Research Centre
PIM2: Paediatric Index Mortality 2 score
DAG: Directed Acyclic Graph
AUC: Area Under the Curve

## References

[1] Pearson G, Shann F, Barry P, Vyas J, Thomas D, Powell C (1997). Should paediatric intensive care be centralised? Trent versus Victoria. Lancet.

[2] Department of Health (1997). Paediatric intensive care: A framework for the future. Report from the National Coordinating Group on Paediatric Intensive Care to the Chief Executive of the NHS Executive.

[3] Gemke R (1997). Centralisation of paediatric intensive care to improve outcome. Lancet.

[4] Pollack M, Alexander S, Clarker N, Ruttumann U, Tesselaar H, Bachulis A (1991). Improved outcomes from tertiary center pediatric intensive care: a statewide comparison of tertiary and nontertiary care facilities. Crit Care Med.

[5] Ramnarayan P, Polke E (2012). The state of paediatric intensive care retrieval in Britain. Arch Dis Child.

[6] PICANet. Annual Report 2018. Online at: https://www.picanet.org.uk/annual-reporting-and-publications/ [Last accessed: 20/6/2019].

[7] Ramnarayan P, Thiru K, Parslow RC, Harrison DA, Draper ES, Rowan KM (2010). Effect of specialist retrieval teams on outcomes in children admitted to paediatric intensive care units in England and Wales: a retrospective cohort study. Lancet.

[8] Moynihan K, McSharry B, Reed P, Buckley D (2016). Impact of retrieval, distance traveled, and referral center on outcomes in unplanned admissions to a national PICU. Pediatr Crit Care Med.

[9] Orr RA, Felmet KA, Han Y, McCloskey KA, Dragotta MA, Bills DM (2009). Pediatric Specialized Transport Teams Are Associated With Improved Outcomes. Pediatrics.

[10] Paediatric Intensive Care Society (2015). Quality standards for the care of critically ill children.

[11] NHS England. PICU Quality Dashboard. https://www.england.nhs.uk/wp-content/uploads/2018/03/picu-metric-definitions-2018-19.pdf [Last accessed: 3/9/19].

[12] Bigham MT, Schwartz HP (2013). Quality metrics in neonatal and pediatric critical care transport: a consensus statement. Pediatr Crit Care Med.

[13] Schwartz HP, Bigham MT, Schoettker PJ, Meyer K, Trautman MS, Insoft RM (2015). Quality metrics in neonatal and pediatric critical care transport: a National Delphi Project. Pediatr Crit Care Med.

[14] Rollin A (2006). Working together for the sick or injured child: the Tanner report. Anaesthesia.

[15] Ramnarayan P, Evans R, Draper ES, Seaton SE, Wray J et al. Differences in access to Emergency Paediatric Intensive Care and care during Transport (DEPICT): study protocol for a mixed methods study. BMJ Open. 2019;9:e028000. 10.1136/bmjopen-2018-028000. 10.1136/bmjopen-2018-028000 PMC6661595 31315865

[16] Slater A, Shann F, Pearson G (2003). PIM2: a revised version of the Paediatric index of mortality. Intensive Care Med.

[17] Zou KH, O'Malley AJ, Mauri L (2007). Receiver-Operating Characteristic Analysis for Evaluating Diagnostic Tests and Predictive Models. Circulation.

[18] Hosmer DW, Lemeshow S (2013). Applied logistic regression.

[19] Brier GW (1950). Verification of forecasts expressed in terms of probability. Mon Weather Rev.

[20] Davis AL, Carcillo JA, Aneja RK, Deymann AJ, Lin JC, Nguyen TC (2017). The American College of Critical Care Medicine Clinical Practice Parameters for hemodynamic support of pediatric and neonatal septic shock: executive summary. Pediatr Crit Care Med.

[21] Kochanek PM, Tasker RC, Carney N, Totten AM, Adelson PD, Selden NR (2019). Guidelines for the Management of Pediatric Severe Traumatic Brain Injury, third edition: update of the brain Trauma Foundation guidelines, Executive Summary. Pediatric Critical Care Med.

[22] Vos GD, Nissen AC, Nieman FHM, Meurs MM, van Waardenburg DA, Ramsay G (2004). Comparison of interhospital pediatric intensive care transport accompanied by a referring specialist or a specialist retrieval team. Intensive Care Med.

[23] King M, Ramnarayan P, Seaton SE, Pagel C (2019). Modelling the allocation of paediatric intensive care retrieval teams in England and Wales. Arch Dis Child.

[24] Ramnarayan P, Patel K, Pappachan J, Purday J, Davis P, Harrison D (2013). Characteristics and outcome of children admitted to adult intensive care units in England, Wales and Northern Ireland (1996–2011). Intensive Care Med.

[25] Borrows EL, Lutman DH, Montgomery MA, Petros AJ, Ramnarayan P (2010). Effect of patient- and team-related factors on stabilization time during pediatric intensive care transport. Pediatr Crit Care Med.

