# Supplementary figures and images for: Does time taken by paediatric critical care transport teams to reach the bedside of critically ill children affect survival? A retrospective cohort study from England and Wales
Source: BMC Pediatr. 2020 Jun 19;20:301. doi: 10.1186/s12887-020-02195-6 (PMC7304220; doi:10.1186/s12887-020-02195-6)

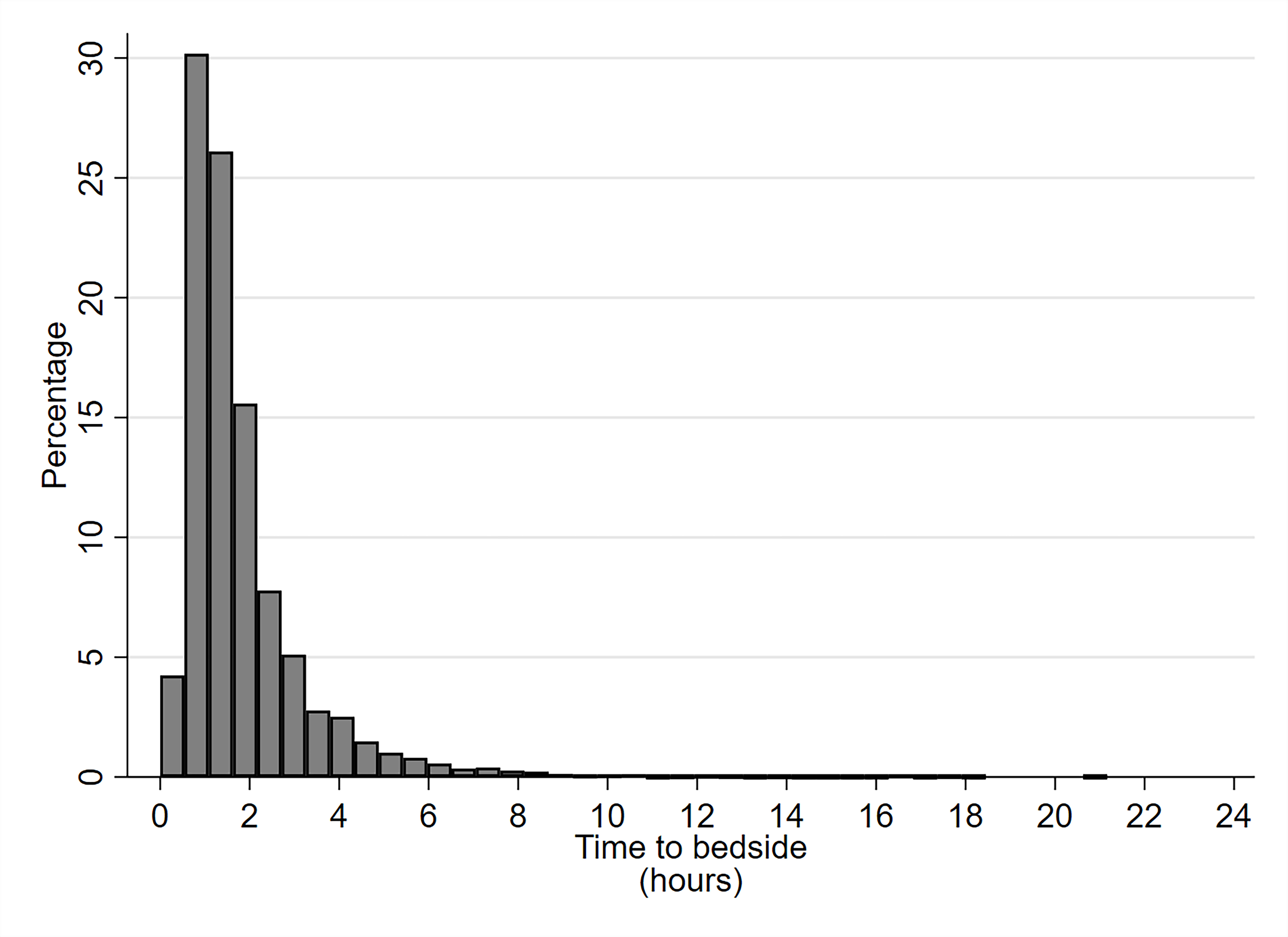

Supplement: Supplementary file 1 — Additional file 1: Figure 1: Distribution of time taken to reach the bedside in the hospital requesting paediatric transport (n = 9116). [file 12887_2020_2195_MOESM1_ESM.jpg]

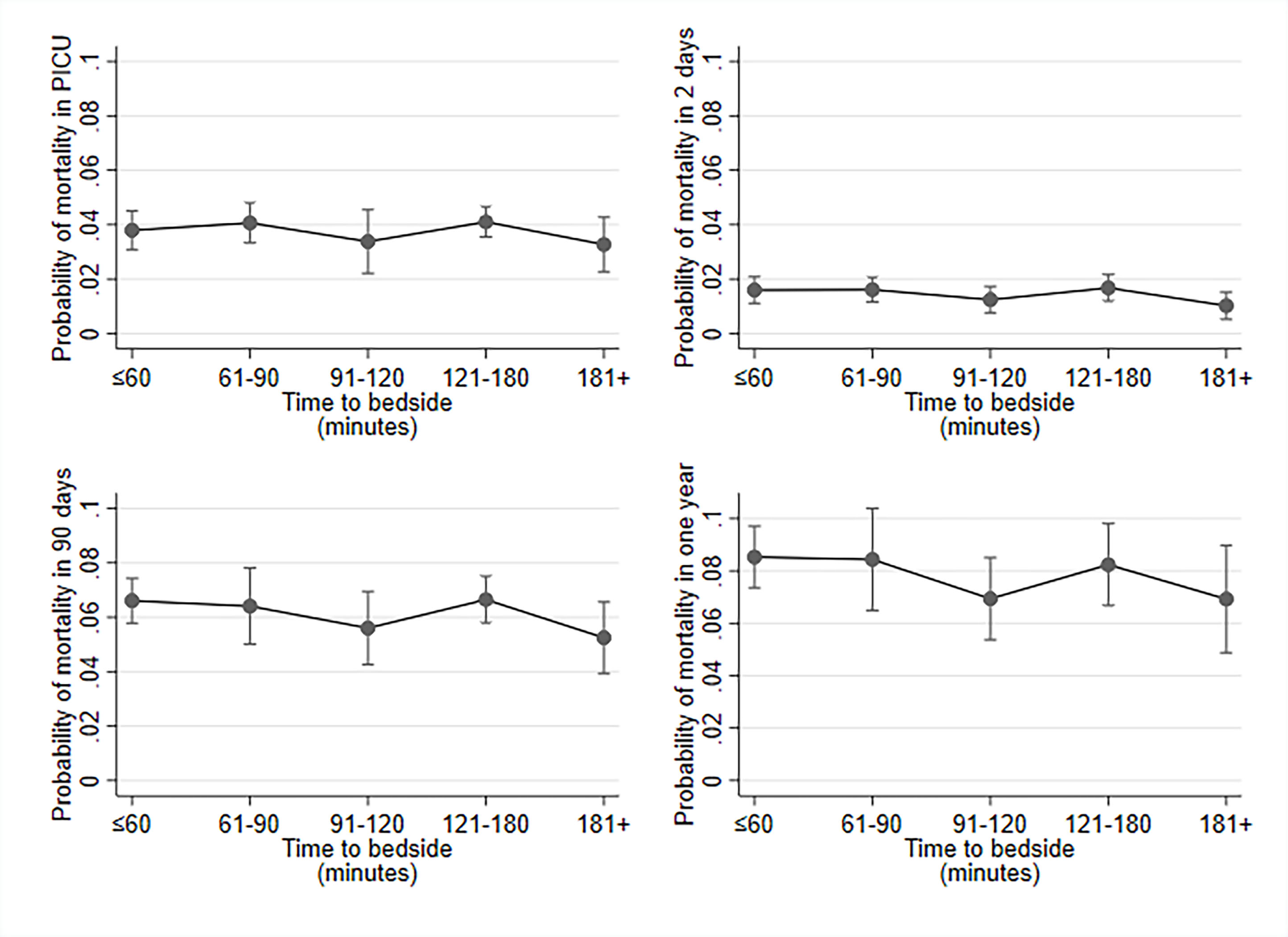

Supplement: Supplementary file 2 — Additional file 2: Figure 2: Mortality in the PICU, in two days, 90 days and within one year of admission against time taken to reach the bedside whilst holding other variables in the model at the mean value. [file 12887_2020_2195_MOESM2_ESM.jpg]

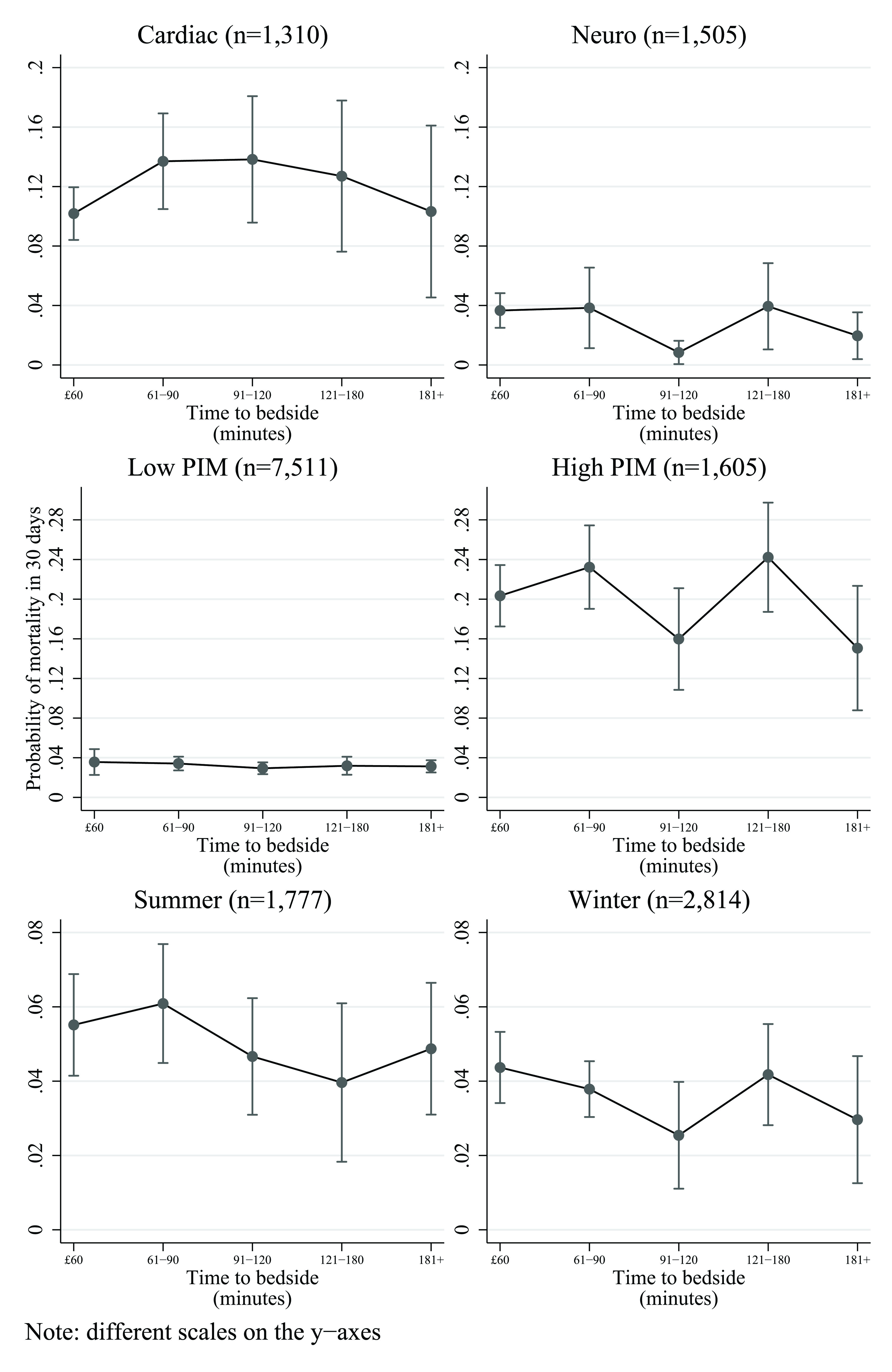

Supplement: Supplementary file 3 — Additional file 3: Figure 3: Subgroup analysis for time taken to reach the bedside on mortality within 30 days of admission to PICU. Adjustments are as in the primary analysis. [file 12887_2020_2195_MOESM3_ESM.jpg]
